# Nonlinear association of triglyceride‐glucose body mass index with all‐cause mortality in postmenopausal women, a retrospective cohort study: Evidence from the 2001 to 2014 National Health and nutrition examination survey

**DOI:** 10.1002/ijgo.70778

**Published:** 2025-12-31

**Authors:** Xiliang Wang, Kaiyue Wang, Chang Tan, Yuexin Yu

**Affiliations:** ^1^ Department of Reproductive Medicine General Hospital of Northern Theater Command Shenyang China

**Keywords:** 2001 to 2014 National Health and nutrition examination survey, all‐cause mortality, nonlinear association, postmenopausal women, triglyceride glucose‐body mass index

## Abstract

**Background:**

The triglyceride‐glucose body mass index (TyG‐BMI) is a novel indicator combining an insulin resistance proxy (TyG index) with adiposity. It remains unclear how TyG‐BMI relates to long‐term mortality risk in postmenopausal women, a group prone to metabolic changes after menopause. This study investigated the association between TyG‐BMI and all‐cause mortality, with secondary outcomes of cardiovascular disease (CVD) mortality and cancer mortality, in a nationally representative cohort of US postmenopausal women.

**Methods:**

We analyzed data from 3844 postmenopausal women (mean age 64.8 ± 10.8 years) in the 2001–2014 National Health and Nutrition Examination Survey (NHANES) who had baseline fasting triglycerides, glucose, and body mass index (BMI) measured. The TyG‐BMI index was calculated as ln[fasting triglycerides (mg/dL) × fasting glucose (mg/dL)/2] × BMI (kg/m^2^). Mortality outcomes (all‐cause, CVD, and cancer mortality) were ascertained through linkage to National Death Index records, with median follow‐up of 119 months (approximately 9.9 years). Cox proportional hazards models were used to estimate hazard ratios (HRs) for mortality per standard deviation (SD) increase in TyG‐BMI, adjusting for age, ethnicity, poverty‐income ratio, smoking, alcohol use, and total physical activity (MET‐min/week). We used restricted cubic splines to assess nonlinearity and a two‐piece Cox model to estimate threshold effects, with log‐likelihood ratio tests to confirm nonlinear associations. Sensitivity analyses were conducted to test the robustness of findings.

**Results:**

Over follow‐up, 1033 participants died (26.9%), including 336 CVD deaths (8.7%) and a similar order of magnitude of cancer deaths. The baseline TyG‐BMI averaged 260.7 ± 67.0. We observed a nonlinear (U‐shaped) relationship between TyG‐BMI and all‐cause mortality (*P* < 0.0001 for nonlinearity). An inflection point was identified at a TyG‐BMI of approximately 289. Below this point, higher TyG‐BMI was associated with lower mortality risk: for each 1 SD (67.0‐unit) increase in TyG‐BMI, the adjusted HR for all‐cause mortality was 0.77 (95% confidence interval [CI] 0.69–0.86, *P* < 0.0001). Above the threshold of 289, higher TyG‐BMI was associated with higher mortality risk: per SD increase HR = 1.28 (95% CI 1.13–1.46, *P* = 0.0002). In secondary outcome analyses, TyG‐BMI showed a similar nonlinear pattern with CVD mortality (U‐shaped association, with low TyG‐BMI linked to higher CVD death risk and an increasing hazard at the upper end), although estimates were less precise due to fewer events. By contrast, no significant positive association was observed between TyG‐BMI and cancer mortality; if anything, higher TyG‐BMI tended to correspond to a slight reduction in cancer mortality risk, although this trend did not reach statistical significance. All results were consistent in sensitivity analyses.

**Conclusion:**

In this cohort of US postmenopausal women, TyG‐BMI exhibited a U‐shaped association with all‐cause mortality. Both low and high TyG‐BMI values conferred elevated mortality risk, especially from cardiovascular causes, while intermediate TyG‐BMI levels were associated with the lowest risk. These findings suggest that maintaining a balanced TyG‐BMI, reflecting neither extreme leanness with low metabolic reserves nor excessive insulin‐resistant adiposity, might be important for longevity in older women.

## 1 INTRODUCTION

Menopause is a critical transition that often leads to adverse metabolic changes in women, including weight gain, redistribution of body fat, and increased insulin resistance.^[^ ^1^ ^]^ Postmenopausal women consequently face elevated risks of cardiovascular disease (CVD) and other chronic conditions.^[^ ^2^, ^3^ ^]^ Identifying metabolic risk markers that predict mortality in this population could improve risk stratification and preventive strategies. Over the past two years, the triglyceride‐glucose (TyG) index, calculated from fasting triglyceride and glucose levels, has gained attention as a simple surrogate of insulin resistance.^[^ ^4^ ^]^ The TyG index correlates strongly with insulin resistance as measured by hyperinsulinemic‐euglycemic clamps and outperforms the traditional HOMA‐IR in some populations.^[^ ^5^, ^6^ ^]^ Elevated TyG index has been linked to higher risks of type 2 diabetes and CVD events.^[^ ^7^ ^]^

Building on the TyG index, researchers have proposed combined metrics that incorporate anthropometric factors to capture overall metabolic risk. One such metric is the TyG‐BMI index, which multiplies the TyG index by body mass index (BMI). The TyG‐BMI index is intended to integrate dyslipidemia, glycemia, and adiposity into a single indicator. Recent studies suggest TyG‐BMI is strongly correlated with insulin resistance and might be a feasible tool for early metabolic risk assessment.^[^ ^8^ ^]^ For instance, Yan et al. (2023) found TyG‐BMI to have high diagnostic value for insulin resistance in US adults.^[^ ^9^ ^]^ Further, TyG‐BMI has been associated with various cardiometabolic conditions, including heart failure, fatty liver disease, and stroke.^[^ ^10^, ^11^, ^12^ ^]^ However, there is ongoing debate about whether TyG‐BMI can predict long‐term outcomes such as mortality, especially in non‐diabetic general populations. Most prior research on TyG‐related indices and mortality has focused on patients with diabetes or other high‐risk conditions. The present study aims to address this gap by examining the relationship between TyG‐BMI and all‐cause and cause‐specific mortality in postmenopausal women from a nationally representative US cohort. We hypothesized that extreme values of TyG‐BMI (either too low or too high) would be associated with higher mortality risk, potentially reflecting a U‐shaped association. Given that both metabolic derangements (e.g., insulin resistance and obesity) and frailty/malnutrition are relevant concerns in older women, a nonlinear relationship between TyG‐BMI and mortality is biologically plausible.

## 2 METHODS

### 2.1 Study population

**FIGURE 1:**
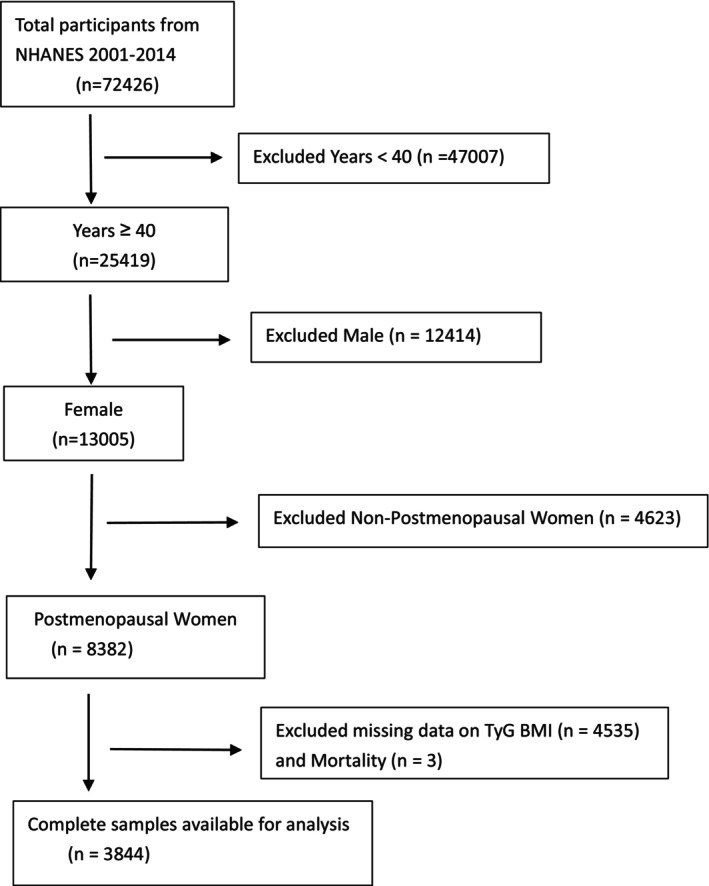
Flow chart of the sample selection from NHANES 2001–2014. NHANES, National Health and Nutrition Examination Survey; TyG‐BMI, triglyceride‐glucose body mass index.

This study is a retrospective cohort study that utilizes data from the US National Health and Nutrition Examination Survey (NHANES) for the years 2001–2014, with mortality follow‐up data through 2015. NHANES is an ongoing, nationally representative survey of the noninstitutionalized US population conducted through household interviews and standardized examinations at Mobile Examination Centers (MECs). It uses a stratified multistage probability sampling design and is fielded biennially; the present analysis therefore uses data collected via both interviews and MEC examinations.^[^ ^13^ ^]^ Participants undergo interviews, physical examinations, and laboratory tests. For the present analysis, we included women who were postmenopausal at the time of their NHANES examination. Postmenopausal status was defined as self‐reported absence of menstrual periods for at least 12 consecutive months (natural menopause) or having undergone bilateral oophorectomy/menopause‐inducing surgery. Among women in the 2001–2014 NHANES cycles, we further required availability of fasting plasma glucose and triglyceride measurements (collected in the morning session after fasting) and BMI data because these are needed to calculate the TyG‐BMI index. TyG‐BMI was evaluated only once at baseline (i.e., using measurements obtained at the index NHANES examination); repeat measurements were not used. We excluded individuals with missing data on key covariates or outcomes. Specifically, participants were excluded if aged <40 years at baseline, they had missing values for fasting triglycerides, fasting plasma glucose, measured weight or height (required for BMI), self‐reported menopausal status, mortality linkage, or any covariate required for the fully adjusted model (refer to the section below on covariates). The final analytic sample consisted of 3844 postmenopausal women aged approximately 40–85 years at baseline. All participants provided written informed consent, and the NHANES protocol was approved by the National Center for Health Statistics Research Ethics Review Board (Protocol #98–12, Protocol #2005–06, and Protocol #2011–17).^[^ ^14^ ^]^ This study was determined to be exempt from additional institutional review board review as it involves secondary analysis of de‐identified public data (Figure 1).

### 2.2 Exposure measurement: Triglyceride‐glucose body mass index

Fasting triglycerides and glucose were measured using standardized assays at NHANES mobile examination centers (triglycerides by enzymatic methods, and glucose by a hexokinase method).^[^ ^15^ ^]^ BMI was calculated as weight in kilograms divided by height in meters squared, measured during the physical exam. We computed the TyG‐BMI index for each participant using the following formula: TyG‐BMI = ln [TG (mg/dL) × FPG (mg/dL)/2] × BMI (kg/m2).^[^ ^16^ ^]^ This formula yields a continuous value reflecting combined lipid, glycemic, and adiposity status. In our study population, TyG‐BMI values ranged broadly. For descriptive purposes, we report TyG‐BMI both as a continuous variable (mean and standard deviation) and in tertiles. For categorical analyses, TyG‐BMI tertiles were generated using the unweighted distribution of TyG‐BMI in the analytic sample (i.e., cut‐points corresponding to the 33.3rd and 66.7th percentiles of the observed values unless otherwise stated); when weighted tertiles were used for sensitivity checks, we explicitly noted this in the results.

### 2.3 Outcome assessment: Mortality

The primary outcome was all‐cause mortality. Secondary outcomes were death from CVD (CVD mortality) and death from malignant neoplasms (cancer mortality). Mortality status and cause of death were determined by linking NHANES participant records to the National Death Index (NDI) through December 31, 2015 (the latest available follow‐up for the 2014 NHANES cohort at the time  of analysis). The duration of follow‐up was calculated from the NHANES examination date to the date of death or end of follow‐up (whichever came first), with a median follow‐up time of 119 months (approximately 9.9 years) in our sample. Cause‐specific mortality was classified using International Classification of Diseases, 10th Revision (ICD‐10) underlying cause codes. Consistent with prior studies,^[^ ^17^ ^]^ we defined CVD mortality as deaths with ICD‐10 codes I00–I09, I11, I13, I20–I51, or I60–I69 (covering heart diseases, hypertensive disease, and cerebrovascular causes). Cancer mortality was defined as deaths with an underlying cause of malignant neoplasm (ICD‐10 codes C00–C97). All‐cause mortality encompassed deaths from any cause. Mortality data were obtained via the publicly available NHANES Linked Mortality File, which is generated by probabilistic matching to NDI records by the National Center for Health Statistics.

### 2.4 Covariates

We included a range of baseline covariates known or suspected to confound the relationship between metabolic factors and mortality. Demographic covariates were age (years) and race/ethnicity (categorized as Non‐Hispanic White, Non‐Hispanic Black, Hispanic, or Other races). Socioeconomic status was represented by the poverty‐to‐income ratio (PIR), a ratio of family income to the federal poverty threshold, categorized in this analysis as <1.0 (below poverty line), 1.0–3.0, or >3.0 (upper income). Health behavior covariates included smoking status (never, former, or current smoker) and alcohol use. Alcohol use was classified into four categories: heavy drinker (on average ≥3 drinks/day for women or frequent binge drinking), moderate drinker, light drinker, or non‐drinker, using NHANES questionnaire definitions.^[^ ^18^ ^]^ We also accounted for physical activity, summarized as total metabolic equivalent (MET) minutes of physical activity per week, combining leisure and work activity. This was treated as a continuous variable or categorized into tertiles for adjustment. Additionally, we adjusted for *baseline comorbidities* that might influence both metabolic status and mortality risk, including self‐reported history of CVD (any coronary heart disease, myocardial infarction, angina, stroke, or heart failure) and history of diabetes because these conditions could affect TyG‐BMI and mortality. (Comorbidity data were obtained via medical condition questionnaires.^[^ ^19^ ^]^) Finally, to partly capture nutritional status beyond BMI, we adjusted for baseline serum albumin and C‐reactive protein in a sensitivity analysis (as proxies for malnutrition/inflammation), although these were not included in the primary models to avoid overadjustment.

### 2.5 Statistical analysis

All analyses accounted for the complex survey design of NHANES, incorporating sample weights, stratification, and clustering, to yield results generalizable to the US population. We applied the 12‐year combined MEC fasting sample weights according to NHANES guidance (using the fasting subsample weight variable appropriate for pooled cycles), and accounted for strata and PSU variables (SDMVSTRA and SDMVPSU) in all survey analyses. We first described baseline characteristics of the participants across tertiles of TyG‐BMI, using means (± standard deviation [SD]) or percentages, and tested differences using one‐way analysis of variance for continuous variables and χ‐tests for categorical variables.

For the primary analysis, we used Cox proportional hazards regression to examine the association between TyG‐BMI (continuous) and time to death. We verified that the proportional hazards assumption was satisfied through Schoenfeld residual tests. Three models were constructed: Model 1 adjusted for age only; Model 2 adjusted for age and race/ethnicity; Model 3 adjusted for the full set of covariates listed above (age, ethnicity, PIR, smoking, alcohol, physical activity, and baseline comorbidities). Because TyG‐BMI was approximately normally distributed, we standardized it to per one SD increase (67.0 units) to facilitate interpretation of HRs. We also assessed TyG‐BMI in tertiles to check for any threshold effects non‐parametrically. Tertiles reported in the main analyses are based on the unweighted sample distribution; sensitivity analyses using survey‐weighted tertiles produced materially similar results.

To formally test for a nonlinear relationship between TyG‐BMI and mortality, we employed restricted cubic spline functions within the Cox models.^[^ ^20^ ^]^ We placed knots at percentile cut‐points (e.g. 5th, 35th, 65th, 95th) and tested the nonlinearity by the significance of the spline terms. Upon observing a significant nonlinear association (*P* < 0.0001), we used a two‐piecewise Cox regression approach (also known as a threshold or piecewise linear model) to estimate effects below and above an optimal inflection point.^[^ ^21^ ^]^ The inflection point (approximately 289) was identified using an iterative maximum‐likelihood algorithm that searched candidate TyG‐BMI values across the observed distribution to find the value that maximized the model log‐likelihood; this candidate value was then rounded to the nearest integer for reporting. We also inspected the spline plot visually to confirm that the chosen threshold corresponded to the apparent change in slope. The inflection point was determined by inspecting the spline curve and using an iterative algorithm to find the TyG‐BMI value at which the model log‐likelihood was maximized.^[^ ^22^ ^]^ A log‐likelihood ratio test comparing the two‐piecewise model to a single‐linear‐term model was performed to confirm a significantly better fit.^[^ ^23^ ^]^ We then computed HRs for a one SD increase in TyG‐BMI below and above the identified threshold.

For secondary outcomes (CVD mortality and cancer mortality), similar Cox models and spline analyses were conducted. However, given the smaller number of events, these analyses might have been underpowered to detect modest nonlinear patterns, so results were interpreted with caution.

We conducted several sensitivity analyses to assess robustness: (1) additionally adjusting for BMI alone (to see if TyG‐BMI offers independent information beyond BMI), (2) restricting to those with at least 2 years of follow‐up (excluding early deaths), and (3) excluding participants with a history of cancer at baseline (to avoid the potential confounding effect of low BMI due to pre‐existing cancer). The two‐sided alpha level was set at 0.05. All the statistical analyses were performed using EmpowerStats (www.empowerstats.com, X&Y solutions, Boston, MA) and R software version 4.2.0 (www.r‐project.org). Key R packages used include survival (version 3.2–13) for Cox models, rms (version 6.3–0) for restricted cubic splines and derivation of knot positions, survey (version 4.1–1) for complex survey weighting, and segmented or flexsurv packages for piecewise modeling; code to reproduce the main models is available upon request.

## 3 RESULTS

### 3.1 Participant characteristics

A total of 3844 postmenopausal women were included, with a mean age of 64.88 years (SD 10.8, range approximately 40–85 years) (Table 1).

**TABLE 1:** Baseline characteristics of participants by TyG‐BMI tertiles (NHANES 2001–2014), weighted.

Characteristics	Overall (114.52–585.42)	Tertile of TyG‐BMI index			*P*‐value
		Low (114.52–225.68)	Middle (225.69–279.16)	High (279.17–585.42)	
*N* (%)	3844	1281 (33.32)	1281 (33.32)	1282 (33.35)	
Age (years)	62.77 (62.30, 63.24)	63.47 (62.70, 64.24)	63.46 (62.74, 64.17)	61.30 (60.60, 62.01)	<0.001
Height (cm)	161.00 (160.69, 161.31)	161.42 (160.96, 161.88)	160.67 (160.15, 161.18)	160.86 (160.28, 161.44)	0.0878
Weight (kg)	76.12 (75.27, 76.97)	60.26 (59.63, 60.89)	73.87 (73.29, 74.45)	96.26 (94.92, 97.59)	<0.001
Body mass index (kg/m^2^)	29.33 (29.02, 29.64)	23.08 (22.90, 23.27)	28.56 (28.41, 28.71)	37.15 (36.71, 37.59)	<0.001
Waist (cm)	98.41 (97.70, 99.11)	84.44 (83.90, 84.99)	98.11 (97.62, 98.60)	114.61 (113.80, 115.41)	<0.001
TyG‐BMI	258.02 (254.77, 261.27)	193.72 (192.17, 195.26)	250.95 (249.79, 252.10)	337.65 (333.62, 341.68)	<0.001
Ethnicity					<0.001
Non‐Hispanic White	77.51 (74.93, 79.90)	81.86 (79.06, 84.36)	75.16 (71.56, 78.45)	74.93 (71.41, 78.15)	
Non‐Hispanic Black	9.54 (8.21, 11.06)	6.20 (4.95, 7.73)	10.48 (8.69, 12.58)	12.38 (10.36, 14.72)	
Mexican American	4.56 (3.57, 5.80)	2.64 (1.98, 3.53)	5.13 (3.95, 6.62)	6.16 (4.66, 8.10)	
Other Hispanic	3.55 (2.72, 4.64)	2.74 (1.93, 3.88)	5.17 (3.70, 7.19)	2.87 (2.09, 3.91)	
Other race	4.84 (3.95, 5.91)	6.56 (5.18, 8.27)	4.06 (2.87, 5.72)	3.67 (2.40, 5.56)	
Marital					0.0396
Married/Living with partner	59.89 (58.17, 61.58)	61.87 (58.48, 65.15)	60.06 (56.65, 63.38)	57.47 (54.13, 60.75)	
Widowed/Divorced/Separated	34.98 (33.22, 36.77)	34.69 (31.40, 38.13)	34.87 (31.72, 38.16)	35.40 (31.99, 38.97)	
Never married	5.13 (4.23, 6.22)	3.44 (2.51, 4.70)	5.05 (3.43, 7.38)	7.12 (5.57, 9.07)	
Missing	0.00 (0.00, 0.03)	0.00 (0.00, 0.00)	0.01 (0.00, 0.09)	0.00 (0.00, 0.00)	
Education level					<0.001
Below high school	7.27 (6.40, 8.25)	5.25 (4.14, 6.65)	9.04 (7.47, 10.90)	7.78 (6.32, 9.55)	
High school	39.07 (36.85, 41.34)	34.03 (30.75, 37.46)	40.98 (37.23, 44.83)	42.87 (38.82, 47.02)	
Above high school	53.64 (51.24, 56.02)	60.68 (57.14, 64.11)	49.95 (46.09, 53.83)	49.35 (44.94, 53.76)	
Missing	0.02 (0.01, 0.06)	0.04 (0.01, 0.14)	0.03 (0.01, 0.11)	0.00 (0.00, 0.00)	
Smoking status					0.036
Never	55.51 (53.03, 57.96)	54.01 (50.34, 57.63)	57.05 (53.41, 60.61)	55.69 (52.71, 58.63)	
Former	28.62 (26.50, 30.85)	28.06 (25.22, 31.10)	26.94 (23.62, 30.54)	30.92 (28.07, 33.92)	
Now	15.81 (13.79, 18.06)	17.90 (14.36, 22.09)	15.90 (13.43, 18.72)	13.37 (11.21, 15.86)	
Missing	0.06 (0.02, 0.20)	0.03 (0.00, 0.21)	0.12 (0.02, 0.68)	0.02 (0.00, 0.18)	
Alcohol use					<0.001
Never	19.14 (17.41, 21.01)	15.63 (13.48, 18.06)	22.45 (19.54, 25.66)	19.82 (17.09, 22.87)	
Former	23.12 (21.33, 25.01)	20.32 (17.60, 23.33)	20.54 (17.84, 23.54)	28.84 (25.11, 32.89)	
Mild	34.58 (32.03, 37.21)	39.94 (35.96, 44.06)	33.21 (29.90, 36.69)	29.87 (26.54, 33.43)	
Moderate	15.61 (14.00, 17.36)	16.13 (13.75, 18.84)	15.81 (13.05, 19.03)	14.81 (12.01, 18.13)	
Heavy	7.38 (6.19, 8.77)	7.79 (5.86, 10.28)	7.95 (6.22, 10.10)	6.34 (4.93, 8.11)	
Missing	0.18 (0.07, 0.46)	0.19 (0.06, 0.55)	0.03 (0.00, 0.25)	0.32 (0.09, 1.05)	
Total physical activity (MET/week)					<0.001
>600	27.73 (25.87, 29.66)	27.91 (24.61, 31.47)	26.88 (23.49, 30.55)	28.36 (25.73, 31.15)	
≥ 600	39.72 (37.48, 42.01)	47.09 (43.29, 50.93)	41.33 (37.28, 45.50)	29.80 (26.91, 32.86)	
Missing	32.55 (30.53, 34.64)	25.00 (22.13, 28.10)	31.80 (28.60, 35.17)	41.84 (38.51, 45.23)	
Hypertension					<0.001
No	40.21 (38.23, 42.22)	53.28 (49.65, 56.87)	38.31 (34.92, 41.81)	27.33 (23.96, 30.98)	
Yes	59.79 (57.78, 61.77)	46.72 (43.13, 50.35)	61.69 (58.19, 65.08)	72.67 (69.02, 76.04)	
Diabetes mellitus					<0.001
No	59.76 (57.30, 62.17)	78.18 (74.94, 81.10)	58.87 (55.55, 62.10)	39.85 (35.90, 43.93)	
Diabetes mellitus	22.77 (20.96, 24.68)	8.92 (7.24, 10.94)	21.98 (19.67, 24.49)	39.18 (35.30, 43.21)	
IFG	8.18 (7.18, 9.31)	4.34 (3.26, 5.77)	9.34 (7.45, 11.66)	11.36 (9.14, 14.03)	
IGT	9.29 (7.99, 10.78)	8.56 (6.67, 10.94)	9.80 (7.95, 12.03)	9.61 (7.58, 12.10)	
Hyperlipidemia					<0.001
No	13.25 (11.75, 14.91)	20.79 (17.89, 24.03)	8.84 (7.18, 10.84)	9.11 (7.39, 11.18)	
Yes	86.75 (85.09, 88.25)	79.21 (75.97, 82.11)	91.16 (89.16, 92.82)	90.89 (88.82, 92.61)	

*Note*: Data are presented as mean ± standard deviation or *n* (%).

Abbreviations: IFG, impaired fasting glycemia; IGT, impaired glucose tolerance; MET, metabolic equivalent; NHANES, National Health and Nutrition Examination Survey; TyG‐BMI, triglyceride‐glucose body mass index.

### 3.2 Mortality outcomes

**FIGURE 2:**
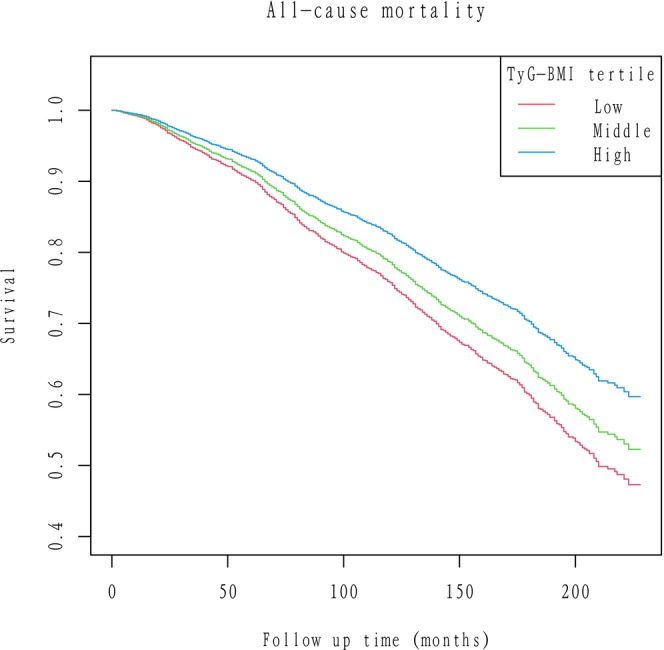
Kaplan–Meier curves of the survival rate and the number (%) of at‐risk postmenopausal women. TyG‐BMI index tertile low was used as the reference group. TyG‐BMI, triglyceride‐glucose body mass index.

During a median follow‐up of 119 months, a total of 1033 participants died from any cause (cumulative mortality 26.9%). Among these, 336 deaths (8.7% of the cohort) were due to cardiovascular causes, and an estimated 212 (approximately 5.5%) were due to cancer (CVD and cancer were the two leading causes of death, together accounting for approximately 53% of all deaths) (Table 2). Kaplan–Meier curves indicated lower all‐cause mortality in patients with elevated TyG‐BMI compared to lower levels (*P* < 0.0001) (Figure 2).

**TABLE 2:** Follow‐up time and mortality outcomes (NHANES 2001–2014).

Follow‐up time (months) Median (Q1–Q3)	119.00 (81.00–159.25)
All‐cause mortality *N* (%)	
Survival	2811 (73.13%)
Non‐survival	1033 (26.87%)
Cardiovascular mortality *N* (%)	
No	3508 (91.26%)
Yes	336 (8.74%)
Cancer mortality *N* (%)	
No	3632 (94.48%)
Yes	212 (5.52%)

Abbreviations: NHANES, National Health and Nutrition Examination Survey; Q1, first quartile; Q3, third quartile.

### 3.3 All‐cause mortality—Nonlinear association with TyG‐BMI

**FIGURE 3:**
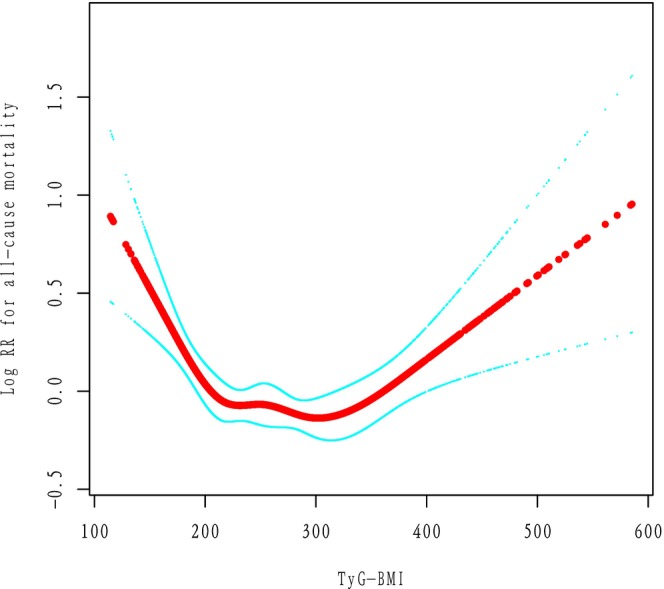
Smoothing curve fitting of TyG‐BMI and all‐cause mortality. Adjusted for age, race/ethnicity, poverty income ratio, smoking, alcohol use, and total physical activity time (min/week). TyG‐BMI, triglyceride‐glucose body mass index.

In fully adjusted Cox models treating TyG‐BMI as a continuous linear term, we found no significant linear association with all‐cause mortality (HR per SD increase = 0.96, 95% CI 0.89–1.03, *P* = 0.26), suggesting that a simple linear model was inadequate (Table 3). However, when we modeled TyG‐BMI with restricted cubic splines, a clear nonlinear pattern emerged (*P* < 0.0001 for the overall spline term) (Figure 3).

**TABLE 3:** Threshold effect analysis of TyG index on all‐cause mortality in postmenopausal women (NHANES 2001–2014), weighted.

All‐cause mortality	TyG‐BMI *Z*‐score
Model I	
Linear effect	0.96 (0.87, 1.05) 0.394
Model II	
Breakpoint (K)	TyG‐BMI = 289
Segment effect 1 (<K)	0.75 (0.63, 0.91) 0.004
Segment effect 2 (>K)	1.31 (1.07, 1.59) 0.007
*P* for log‐likelihood ratio test	<0.001

Abbreviations: NHANES, National Health and Nutrition Examination Survey; TyG, triglyceride‐glucose. TyG‐BMI, triglyceride‐glucose body mass index.

### 3.4 Cardiovascular disease and cancer mortality

**FIGURE 4:**
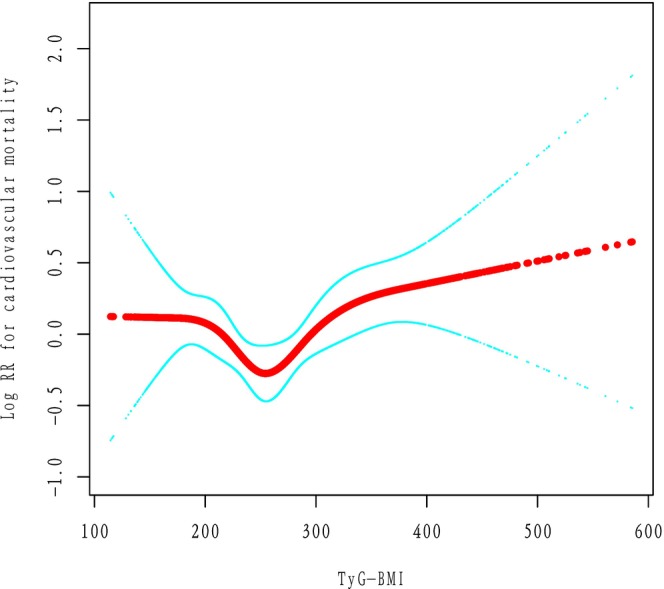
Smoothing curve fitting of TyG‐BMI and cardiovascular mortality. Adjusted for age, race/ethnicity, poverty income ratio, smoking, alcohol use, and total physical activity time (min/week). TyG‐BMI, triglyceride‐glucose body mass index.

For cardiovascular mortality and cancer mortality, we observed a broadly similar pattern. The spline analysis suggested nonlinearity (*P* ≈ 0.01 for spline), with a possible risk nadir around a TyG‐BMI in the high‐200 s (close to the threshold for all‐cause) (Figure 4) (Supplement 1).

### 3.5 Sensitivity and subgroup analyses

**FIGURE 5:**
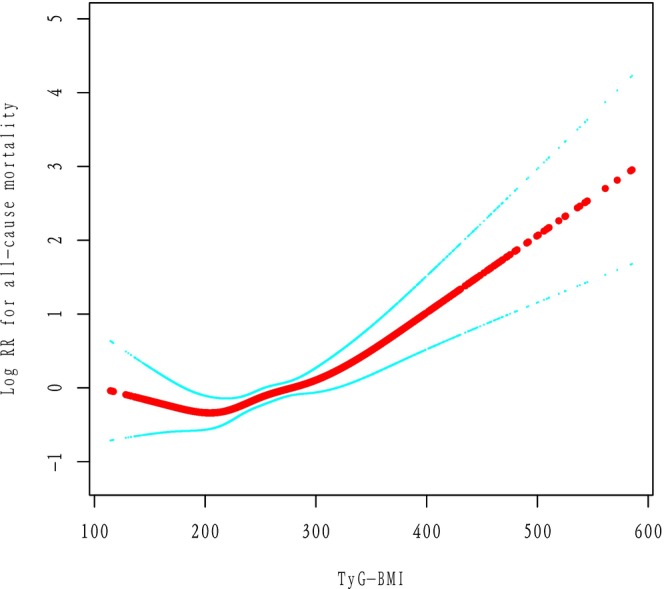
Smoothing curve fitting of TyG‐BMI and all‐cause mortality with additional adjustment for BMI. Adjusted for age, race/ethnicity, poverty income ratio, smoking, alcohol use, total physical activity time (min/week) and BMI. BMI, body mass index; TyG‐BMI, triglyceride‐glucose body mass index.

**FIGURE 6:**
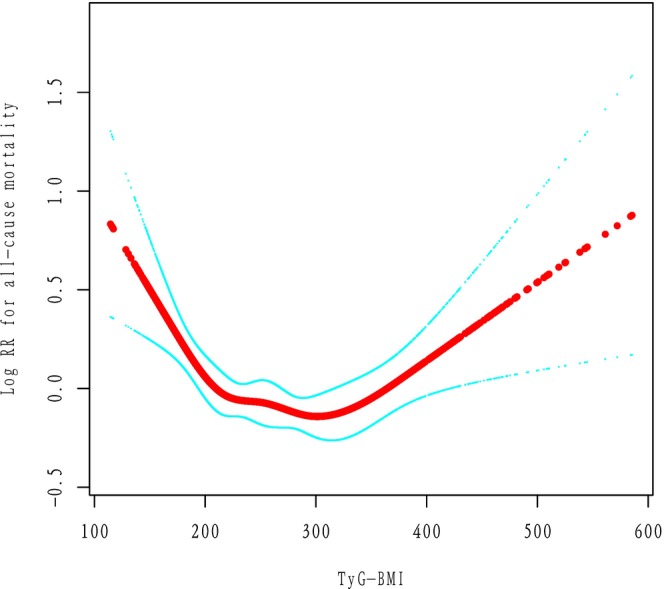
Smoothing curve fitting of TyG‐BMI and all‐cause mortality excluding the first 2 years of follow‐up. Adjusted for age, race/ethnicity, poverty income ratio, smoking, alcohol use, and total physical activity time (min/week). TyG‐BMI, triglyceride‐glucose body mass index.

Our results proved robust in multiple sensitivity analyses. Additional adjustment for BMI (in place of TyG‐BMI's BMI component) attenuated the strength of the associations, but the U‐shape persisted, suggesting TyG‐BMI's predictive ability is not solely due to BMI (Figure 5). Excluding the first 2 years of follow‐up (to reduce influence of undetected serious illness at baseline) actually strengthened the inverse association at low TyG‐BMI, consistent with the idea that baseline frailty (with low BMI and low lipids) confers high short‐term mortality (Figure 6).

## 4 DISCUSSION

In this large prospective study of postmenopausal US women, we found that the TyG‐BMI index had a U‐shaped relationship with all‐cause mortality, with an inflection around TyG‐BMI of approximately 289. Specifically, lower TyG‐BMI values (reflecting leaner body habitus and/or lower glucose and triglyceride levels) were associated with higher mortality risk, while at higher TyG‐BMI values (reflecting obesity with insulin resistance), mortality risk also increased. To our knowledge, this is one of the first studies to demonstrate a nonlinear association between TyG‐BMI and mortality in a general population sample.

Our findings align with and extend previous research in more select populations. For example, Xiao et al. (2024) observed a U‐shaped relationship between TyG‐BMI and mortality in diabetic patients, with low TyG‐BMI (below approximately 280) associated with reduced survival and high TyG‐BMI associated with increased mortality.^[^ ^9^ ^]^ Our study shows that a similar pattern holds in a broader cohort of older women, not limited to those with diabetes. Moreover, the threshold we identified (approximately 289) is remarkably close to that reported in diabetic patients (approximately 280),^[^ ^9^ ^]^ suggesting a potentially generalizable “sweet spot” range of TyG‐BMI that is optimal for survival.

The elevated risk at the low end of TyG‐BMI likely reflects the adverse consequences of frailty, undernutrition, or catabolic states in older adults. A very low TyG‐BMI could be due to low BMI (possible sarcopenia or cachexia) and/or low lipid‐glucose levels (which might indicate poor nutritional intake or underlying disease). Low BMI has consistently been associated with higher mortality in older populations, exhibiting a U‐ or J‐shaped relation with mortality independent of diabetes status.^[^ ^24^, ^25^ ^]^ Our results concur with this “obesity paradox” phenomenon: being too lean in later life can be detrimental.^[^ ^26^ ^]^ Additionally, low fasting triglyceride levels have been identified as a marker of worse outcomes in certain chronic diseases like heart failure.^[^ ^27^ ^]^ This might be because very low cholesterol or triglyceride levels can accompany chronic illness or reflect reduced hepatic production in frail individuals. Low normal glucose levels are generally not considered dangerous in healthy people, but among diabetics aggressive glucose lowering can increase cardiovascular events via hypoglycemia and adrenergic surges.^[^ ^28^ ^]^ In our non‐diabetic majority cohort, a low TyG‐BMI likely captured those with less robust metabolic reserves. It is also plausible that some women in the lowest TyG‐BMI category had subclinical illnesses (e.g., undiagnosed malignancy leading to weight loss) or generally poorer health, confounding the association. We attempted to mitigate this by excluding early follow‐up deaths, yet the association persisted, reinforcing that intrinsically low TyG‐BMI is a risk marker.

At the opposite extreme, high TyG‐BMI signifies the confluence of adiposity and insulin‐resistant metabolism. This condition predisposes individuals to a host of complications, including type 2 diabetes, atherosclerosis, and heart failure.^[^ ^29^ ^]^ Insulin resistance, reflected in an elevated TyG index, contributes to endothelial dysfunction, pro‐inflammatory and pro‐thrombotic states, and ultimately CVD events.^[^ ^30^ ^]^ Obesity exacerbates these issues by promoting hypertension, dyslipidemia, and systemic inflammation. It is therefore not surprising that very high TyG‐BMI was associated with increased mortality, particularly from cardiovascular causes.^[^ ^31^ ^]^ Our findings agree with prior studies that have shown that higher TyG index or TyG‐BMI predicts greater mortality in various populations. For instance, He et al. (2024) reported that in 3.5 million Chinese adults, once the TyG index exceeded approximately 9.8, the hazard of mortality rose sharply (approximately doubling for all‐cause mortality).^[^ ^32^ ^]^ They also found little risk increase below that threshold, consistent with a reverse J‐ or L‐shaped curve. In another cohort of US adults with cardiometabolic syndrome, high TyG index was linked with greater mortality risk.^[^ ^33^ ^]^ Our study specifically adds that in postmenopausal women, who often have unique risk factor profiles (e.g., high prevalence of metabolic syndrome after menopause), TyG‐BMI can identify those at heightened risk: women with very large body size and metabolic perturbation are at a significantly higher risk of death over 10 years. This emphasizes the need for targeted interventions (e.g., weight management, glucose, and lipid control) in that group.

The lack of a positive association between TyG‐BMI and cancer mortality in our results is noteworthy. It suggests that the deleterious impact of obesity‐insulin resistance on mortality is primarily via cardiovascular pathways rather than cancer in this cohort. Indeed, we observed a slight inverse trend, where higher TyG‐BMI correlated with *lower* cancer mortality, although not significantly. A similar observation was made by He et al. (2024), who found a modest negative linear association between TyG index and cancer mortality (HR approximately 0.97).^[^ ^32^ ^]^ One possible explanation is that mild to moderate hyperinsulinemia and higher adiposity in older women might not markedly raise short‐term cancer mortality and could even be associated with certain protective factors (e.g., higher estrogen levels from adipose tissue might protect against some cancers, or obese patients might receive more aggressive care). Conversely, women with very low TyG‐BMI might include those who had preclinical cancers leading to weight loss or those who are frail and less able to withstand oncologic illnesses. It is important to interpret this finding cautiously; it does not imply that metabolic health is unimportant for cancer outcomes, but it underscores that the relationship between metabolic syndrome and cancer is complex and might not translate into increased mortality within a decade of follow‐up. Prior research on obesity and cancer mortality in older adults has also been mixed, with some studies showing an obesity paradox for certain cancers. Our study was not primarily designed to evaluate cancer outcomes, and the number of cancer deaths was relatively limited, so further investigation in larger samples would be beneficial.

### 4.1 Strengths and limitations

Key strengths of our study include the use of a large, nationally representative sample with nearly 10 years median follow‐up, which enhances the generalizability to US postmenopausal women. We had rigorously measured laboratory and anthropometric data, allowing precise calculation of TyG‐BMI and comprehensive follow‐up for cause‐specific mortality. We adjusted for an array of confounders and performed sensitivity analyses to check robustness. The use of flexible spline modeling was also a strength, enabling us to uncover a nuanced nonlinear association that would have been missed by a simple linear analysis.

Our study also has limitations. First, observational design precludes causal conclusions. While TyG‐BMI is associated with mortality, we cannot confirm it as a causative risk factor; unmeasured factors (e.g., genetic predispositions and unmeasured diseases) could be partly responsible. We tried to adjust for major confounders, but residual confounding is possible. Second, TyG‐BMI was measured at baseline only; trajectories of weight and metabolic factors were not accounted for. Some women might have had changes in their TyG‐BMI over the follow‐up (e.g., weight loss due to illness or intentional lifestyle changes), which could affect mortality risk. Capturing time‐updated exposure might refine the association but was not feasible with the data. Third, there is potential reverse causation, especially for the low TyG‐BMI–high mortality link: women who were destined to die (due to underlying illness) might have had lower BMI or altered metabolism at baseline. Our sensitivity analysis excluding early deaths somewhat alleviates this concern but cannot eliminate it entirely. Fourth, cause‐of‐death misclassification is possible; some deaths classified as CVD might have multifactorial causes, and vice versa for cancer. However, the NDI coding is standard and any misclassification would likely bias results toward null if non‐differential with respect to TyG‐BMI. Finally, our focus on postmenopausal women means the findings might not apply to men or to younger women. There is evidence that metabolic risk markers can have sex‐specific associations with outcomes.^[^ ^34^ ^]^ We chose this group because menopause uniformly increases metabolic risk factors,^[^ ^3^ ^]^ but future studies should examine TyG‐BMI–mortality relationships in other demographics as well.

### 4.2 Clinical and public health implications

Our findings highlight that both extremes of metabolic status in older women confer risk. Traditionally, clinicians focus on the dangers of high BMI and metabolic syndrome, which is valid, as we see elevated mortality at high TyG‐BMI. However, this study suggests that being *too* low in weight and metabolic indices is also a red flag in postmenopausal women. It might be a marker of frailty or existing disease that requires medical attention. The TyG‐BMI index might serve as a convenient summary measure: for example, a woman with a very low TyG‐BMI might benefit from a comprehensive geriatric assessment for frailty or occult illness, whereas a woman with a very high TyG‐BMI should receive counseling and interventions for weight loss, diet, and glucose/lipid management to mitigate CVD risk. From a research perspective, TyG‐BMI could be used in risk prediction models for mortality or CVD in older adults, potentially improving identification of high‐risk individuals beyond using BMI or lipid/glucose metrics alone. It also underscores the importance of a balanced metabolic health. Neither insulin‐resistant obesity nor undernutrition is desirable in late life.

## 5 CONCLUSION

In summary, this study demonstrates a clear U‐shaped association between the TyG‐BMI and mortality among postmenopausal women in the United States. Women with either very low or very high TyG‐BMI faced significantly higher all‐cause and cardiovascular mortality over approximately 10 years, whereas those with intermediate TyG‐BMI had the best survival. These results suggest that extremes of metabolic status are detrimental, with low TyG‐BMI likely reflecting frailty or poor health and high TyG‐BMI reflecting obesity‐related insulin resistance; both portend higher mortality.

## AUTHOR CONTRIBUTIONS

Xiliang wang wrote the main manuscript text. Kaiyue Wang performed data analysis. Chang Tan prepared tables. Yuexin Yu wrote the section on data analysis. Xiliang wang and Kaiyue Wang contributed equally to this work. All authors reviewed the manuscript. The authors read and approved the final manuscript.

## CONFLICT OF INTEREST STATEMENT

The authors have no competing interests to declare.

## ETHICS STATEMENT

The study was conducted in accordance with the guidelines set forth in the Declaration of Helsinki and was approved by the Research Ethics Review Board of the National Center for Health Statistics. All NHANES participants provided written informed consent to participate in this study.

## Supporting information


Data S1.

## Data Availability

The data used for this study are available at the NHANES website. (https://www.cdc.gov/nchs/nhanes/index.html).
