# Supplementary material for: Nonlinear association of triglyceride‐glucose body mass index with all‐cause mortality in postmenopausal women, a retrospective cohort study: Evidence from the 2001 to 2014 National Health and nutrition examination survey
Source: Int J Gynaecol Obstet. 2025 Dec 31;173(3):1569–79. doi: 10.1002/ijgo.70778 (PMC13173619; doi:10.1002/ijgo.70778)
Supplement: Supplementary file 1 — Data S1. [file IJGO-173-1569-s001.docx]

**Supplement 1**


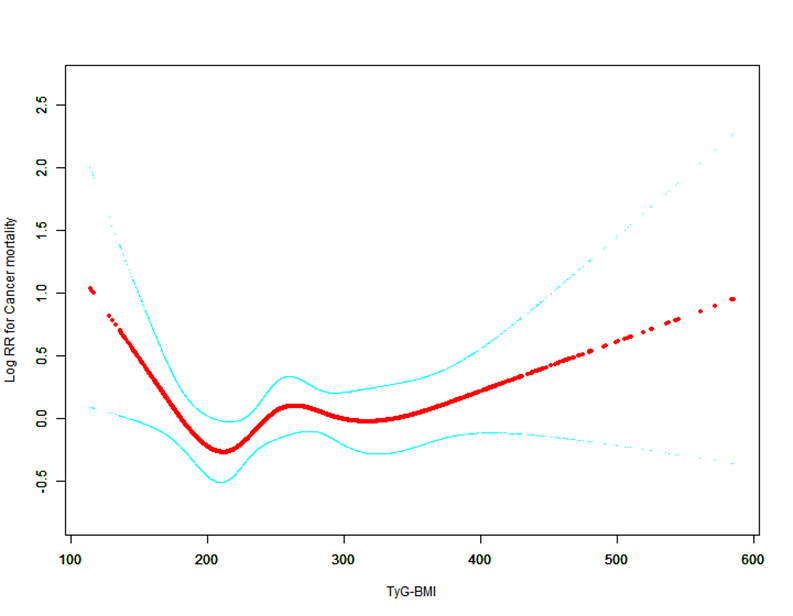


Figure. Smoothing curve fitting of TyG-BMI and cancer mortality. Adjusted for age, race/ethnicity, poverty income ratio, smoking, alcohol use, and total physical activity time(minutes/week). TyG-BMI: Triglyceride-Glucose Body Mass Index.
